# TiO_2_ Nanotube-Enabled Glucose Biosensing: Transformative Insights from 2009 to 2024

**DOI:** 10.3390/mi16111235

**Published:** 2025-10-30

**Authors:** Joydip Sengupta, Chaudhery Mustansar Hussain

**Affiliations:** 1 Department of Electronic Science, Jogesh Chandra Chaudhuri College, Kolkata 700033, India; joydipdhruba@gmail.com; 2 Department of Chemistry and Environmental Science, New Jersey Institute of Technology, Newark, NJ 07102, USA

**Keywords:** glucose biosensor, TiO_2_ nanotubes, enzymatic detection, non-enzymatic sensing, photoelectrochemical interface

## Abstract

The global rise in diabetes has intensified the demand for advanced glucose monitoring technologies that provide continuous, accurate, and real-time detection. Traditional sensing approaches often face challenges related to sensitivity, long-term stability, and suitability for wearable or implantable systems. In this context, titanium dioxide (TiO_2_) nanotube arrays (NTAs) have emerged as a versatile platform owing to their well-defined nanostructure, tunable surface properties, and semiconductor nature, which collectively enable enhanced performance across different sensing modes. These include enzymatic systems, non-enzymatic configurations, and photoelectrochemical (PEC) sensors. While each sensing strategy offers considerable potential, certain inherent limitations continue to be explored. Ongoing research is gradually uncovering various pathways to enhance performance and reliability through the introduction of novel materials and system designs. Looking forward, the broader integration of TiO_2_-based sensing platforms with evolving technological frameworks may contribute to the advancement of more adaptive and user-friendly glucose monitoring solutions.

## 1. Introduction

The global prevalence of diabetes mellitus has reached epidemic proportions, affecting over 537 million adults worldwide and representing one of the most significant public health challenges of the 21st century [1]. This figure is projected to rise to more than 780 million by 2045 [2], underscoring the alarming and sustained increase in diabetes cases year after year. Such an exponential rise not only reflects changes in lifestyle and dietary habits but also highlights the urgent need for early diagnosis and continuous glucose surveillance to effectively manage this chronic disease. This chronic metabolic disorder, characterized by persistent hyperglycemia due to defects in insulin production or action, necessitates continuous glucose monitoring to prevent severe complications including cardiovascular disease [3], nephropathy [4], retinopathy [5], and neuropathy [6]. The critical importance of accurate, reliable, and real-time glucose monitoring has driven intensive research into advanced biosensing technologies that can overcome the limitations of conventional glucose detection methods.

**Figure 1:**
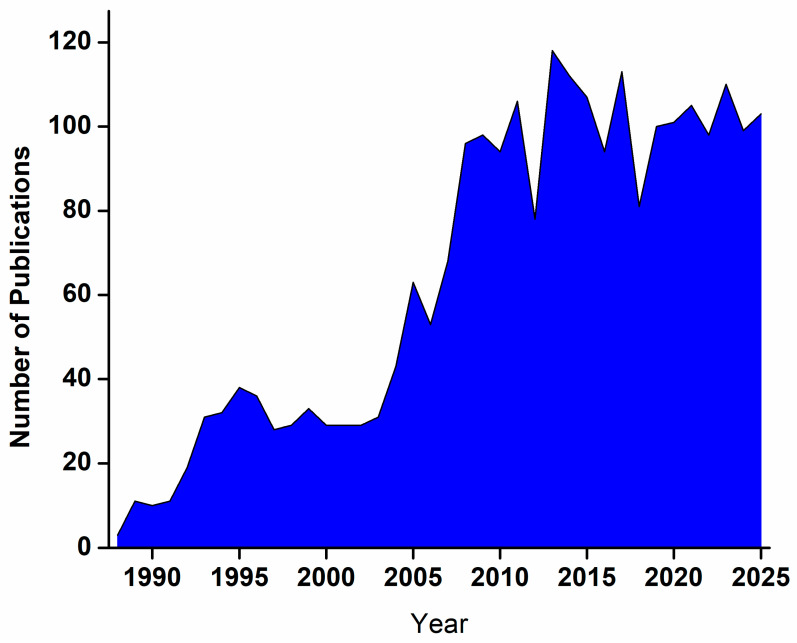
Year-wise distribution of journal articles on glucose biosensors based on data from Scopus, Elsevier.

Conventional glucose detection techniques, such as finger-prick blood tests and enzymatic colorimetric assays, although accurate, are invasive, painful, and not suitable for continuous monitoring. As a result, there has been a paradigm shift toward the development of miniaturized, wearable, and portable electrochemical biosensors capable of providing continuous and real-time glucose monitoring [7]. These next-generation devices—integrated into smartwatches [8], contact lenses [9], skin patches [10], and textile-based sensors [11]—enable seamless and non-invasive glucose measurement from biofluids such as sweat, tears, saliva, and interstitial fluid. Such continuous screening technologies have revolutionized diabetes management by enabling trend analysis, early hypoglycemia detection, and personalized feedback through smartphone connectivity and AI-assisted analytics. The advancement of flexible and stretchable electronic materials, combined with nanostructured electrodes, has further enhanced the performance, comfort, and reliability of these wearable systems [12]. Figure 1 shows the year-wise distribution of journal articles on glucose biosensors, based on Scopus (Elsevier) data.

The emergence of nanotechnology has revolutionized the field of biosensing by providing novel materials with unique properties that can address the limitations of conventional detection methods. Among the diverse array of nanomaterials investigated for glucose sensing applications, TiO_2_ nanotubes have garnered exceptional attention due to their remarkable combination of structural, chemical, and electronic properties. These one-dimensional nanostructures exhibit a highly ordered, vertically aligned architecture [13] that provides unprecedented surface area for biomolecule immobilization and enhanced electron transfer kinetics.

The unique properties of TiO_2_ nanotubes that make them particularly attractive for glucose sensing applications include their exceptional biocompatibility [14], chemical stability [15], and tunable surface chemistry [16]. The large surface-to-volume ratio of these nanostructures enables high-density enzyme loading, while their ordered architecture facilitates efficient mass transport and electron transfer processes.

In the context of electrochemical biosensors, TiO_2_-based nanostructures offer several compelling advantages, such as superior charge-transfer efficiency, photocatalytic activity, and compatibility with various transduction platforms [17]. Their inherent stability and low toxicity make them ideal candidates for integration into wearable and implantable biosensors [18]. Moreover, the tunable band gap and surface functionality of TiO_2_ enable improved enzyme immobilization and enhanced signal transduction, bridging the existing research gap between traditional rigid biosensors and flexible, real-time monitoring systems [19].

Additionally, the semiconducting properties of TiO_2_ can be exploited for PEC applications, opening new avenues for light-enhanced glucose detection with improved sensitivity and selectivity. The combination of photoelectrochemical activity and electrochemical sensing not only enhances the signal-to-noise ratio but also reduces interference from coexisting biomolecules—an essential aspect for accurate continuous monitoring. The versatility of TiO_2_ nanotubes has enabled the development of three distinct yet complementary approaches to glucose sensing: enzymatic, non-enzymatic, and PEC detection strategies. The integration of these diverse detection modalities on the TiO_2_ nanotube platform represents a paradigm shift in glucose sensing technology, offering the potential for multifunctional sensors that can adapt to different operating conditions and requirements.

This review explores the advancements in TiO_2_ nanotube-based glucose sensors across enzymatic, non-enzymatic, and PEC modalities, highlighting their structural advantages, fabrication methods, and sensing performance. Beyond their relevance to diabetes management, these platforms hold promise for broader applications in personalized healthcare and wearable diagnostics. Given the rapid increase in global diabetes incidence and the growing emphasis on continuous, non-invasive, and real-time glucose monitoring, TiO_2_ nanotube-based sensors present a powerful solution for next-generation wearable and portable biosensing devices. Their ability to combine flexibility, durability, and high analytical performance positions them as a cornerstone material for future electrochemical biosensor innovations and real-time healthcare monitoring systems.

## 2. Fundamentals of TiO_2_ NTAs

### 2.1. Synthesis

The synthesis of TiO_2_ NTs plays a pivotal role in determining their morphological, structural, and surface properties, all of which are critical for optimizing biosensing performance. While electrochemical anodization remains the dominant fabrication technique due to its scalability and the formation of uniform, vertically aligned arrays, alternative methods such as hydrothermal, solvothermal, template-assisted, and electrospinning techniques offer tailored advantages for specific sensor configurations and integration strategies.

Electrochemical anodization [20] involves the oxidation of titanium substrates in fluoride-rich electrolytes under controlled electrochemical conditions, where parameters like electrolyte composition, pH, applied potential, and processing time significantly influence the dimensions, wall thickness, and crystalline nature of the resulting nanotubes [21]. Despite offering high reproducibility and compatibility with device-level fabrication, the anodized TiO_2_ typically requires subsequent thermal annealing to transition from its as-formed amorphous state to the anatase or rutile crystalline phases that are more suitable for biosensing due to enhanced photoactivity and surface reactivity [22].

Hydrothermal and solvothermal methods provide an alternative bottom-up strategy that enables direct synthesis of crystalline nanotubular structures under moderate temperature and pressure in a sealed autoclave [23]. These processes involve the transformation of titanium precursors in alkaline environments, facilitating the formation of titanate intermediates that can be proton-exchanged and converted to anatase TiO_2_ through acid washing [24]. The choice of solvent system not only affects the crystalline outcome but also modulates surface functionalities, thereby enhancing molecular recognition and specificity in biosensing applications. Moreover, the relatively low processing temperatures of these methods allow better integration with flexible or heat-sensitive substrates [24].

Template-assisted synthesis utilizes pre-structured nanoporous scaffolds, such as anodic aluminum oxide or polymeric membranes, to guide the deposition of TiO_2_ within predefined geometries [25]. After deposition, the sacrificial template is chemically removed, yielding free-standing nanotube arrays with precise control over diameter, alignment, and aspect ratio—features that are particularly advantageous for applications requiring directional transport, spatially resolved detection, or photonic coupling.

Electrospinning offers a solution-based route to fabricate hybrid or composite one-dimensional TiO_2_ nanostructures by generating precursor-polymer nanofibers, which are then thermally treated to remove organic components [26]. Depending on the precursor composition and calcination protocol, the resulting structures can range from hollow nanotubes to solid nanofibers. This method facilitates the incorporation of functional dopants or secondary phases during synthesis, enabling the design of multifunctional sensing platforms with enhanced electrochemical or optical response [27].

The synthesis method governs the morphology and surface characteristics of TiO_2_ nanotubes, directly impacting biosensor performance. Electrochemical anodization remains the most common technique, producing uniform arrays after thermal annealing to enhance crystallinity. Hydrothermal and solvothermal methods enable direct formation of crystalline nanotubes with tunable surface properties suitable for flexible substrates. Template-assisted synthesis provides precise control over geometry and alignment, while electrospinning offers a simple route to hybrid nanostructures with integrated dopants. Together, these approaches allow tailored fabrication of TiO_2_ nanostructures optimized for high-performance biosensing applications.

### 2.2. Properties

The electronic properties of TiO_2_ NTs are central to their function in electrochemical and PEC glucose sensing. As n-type semiconductors with wide band gaps (~3.2 eV for anatase and ~3.0 eV for rutile) [28], TiO_2_ NTs support photoexcitation-induced charge generation, with a valence band derived from O 2p orbitals and a conduction band composed of Ti 3d orbitals [29]. Although their intrinsic electrical conductivity is limited, it can be significantly improved by introducing oxygen vacancies [30], doping with heteroatoms [31], or coupling with conductive nanomaterials [32]. The highly ordered, one-dimensional architecture of NTs facilitates directional and rapid electron transport, reducing charge recombination and improving signal transduction. Upon UV illumination, TiO_2_ NTs generate electron–hole pairs that drive surface redox reactions [33], forming the basis of PEC glucose sensing. Their optical response is governed by crystal phase and morphology, with anatase showing strong UV absorption and nanotube geometry enhancing light harvesting through confinement effects [34]. Surface states, particularly oxygen vacancies, can boost visible light absorption and conductivity but may also introduce recombination centers, necessitating defect optimization [35]. Moreover, the interface between TiO_2_ NTs and biomolecular or nanomaterial components plays a critical role in determining sensor performance by influencing band alignment and interfacial charge transfer kinetics.

### 2.3. Surface Chemistry and Functionalization

The surface chemistry of TiO_2_ NTs plays a pivotal role in determining their performance in glucose sensing applications [36], as it governs the interactions with enzymes, metal nanoparticles, and other functional moieties necessary for signal transduction. The surface is typically rich in hydroxyl groups [37] and oxygen vacancies [38], which serve as reactive sites for functionalization. Surface hydroxyl groups can be exploited for silanization reactions, allowing covalent attachment of organic linkers and subsequent immobilization of enzymes or other biomolecules. The density and distribution of these groups can be tuned through annealing [39] or chemical treatments [40], enabling surface tailoring for specific sensing applications. Oxygen vacancies, on the other hand, function as intrinsic electron donors that improve charge transport and provide catalytically active sites, especially beneficial for non-enzymatic and PEC sensing [41]. However, an excess of vacancies may lead to instability or undesired recombination effects, thus requiring controlled introduction during synthesis.

There are various functionalization strategies, such as adsorption [42], electrodeposition [43], covalent bonding [44], carbodiimide chemistry [45], etc., for the enhancement of TiO_2_ NT properties. The choice of strategy depends on the sensor configuration. For enzymatic glucose sensors, stable and oriented enzyme immobilization is essential to retain catalytic activity. In non-enzymatic sensors, the attachment of electrocatalytic nanoparticles such as Pt or Cu must preserve surface accessibility and conductivity. For PEC sensors, surface functionalization must not hinder light absorption or charge separation, requiring strategies that enhance photocatalytic activity without compromising optical or electronic properties. Hence, rational surface engineering of TiO_2_ NTs is central to achieving sensitive, stable, and application-specific glucose detection.

## 3. TiO_2_-Based Sensors: Types and Working Principles

TiO_2_ nanotube-based sensors offer a multifunctional platform for glucose detection, owing to their high surface area, excellent charge transport, chemical stability, and tunable surface chemistry. Based on the detection mechanism, these sensors are categorized as enzymatic, non-enzymatic, and PEC.

**Figure 2:**
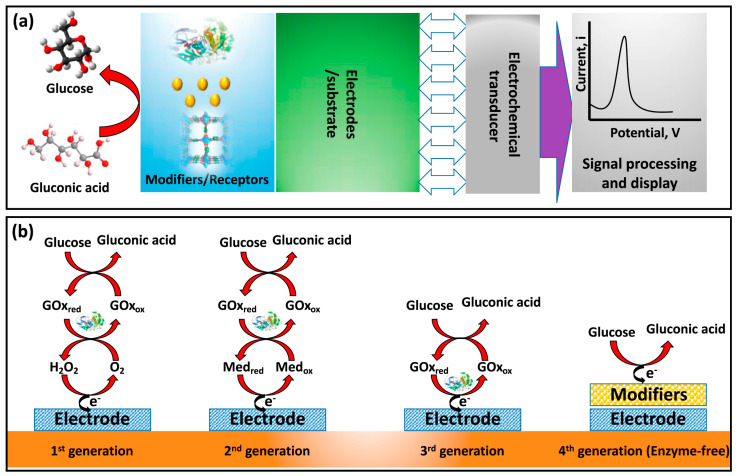
(**a**) Schematic overview and (**b**) operational mechanism illustrating various generations of electrochemical glucose sensors. (Reproduced with permission from [48]).

In enzymatic sensors (Figure 2), TiO_2_ nanotubes act as a scaffold for immobilizing GOx, which catalyzes glucose oxidation, generating H_2_O_2_ [46]. The electrochemical oxidation of H_2_O_2_ produces a measurable current proportional to glucose concentration. TiO_2_ enhances electron transfer and enzyme loading. Further improvements are achieved through surface modification with mediators like AuNPs, Prussian Blue (PB), or carbon nanomaterials, enhancing sensitivity and reducing interferences [47]. Despite high selectivity, enzymatic systems are limited by enzyme instability.

Non-enzymatic sensors (Figure 2) utilize electrocatalytic metals (e.g., Pt, Ni, Cu) anchored onto TiO_2_ nanotubes to directly oxidize glucose, eliminating the need for biological elements [49]. This configuration offers improved stability, longer shelf life, and broad linear response ranges. TiO_2_ ensures uniform nanoparticle dispersion and supports synergistic charge transfer. Bimetallic and transition metal-decorated TiO_2_ sensors exhibit excellent anti-interference capabilities and ultra-low detection limits in complex biofluids [50].

**Figure 3:**
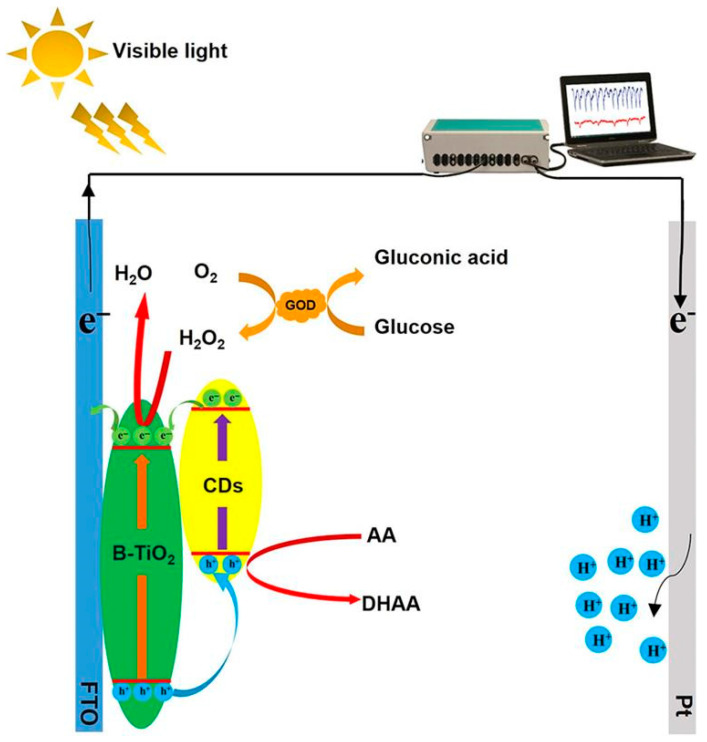
PEC detection mechanism of glucose by CDs/B-TiO_2_/Gox. (Reproduced with permission from [52]).

In PEC glucose sensors (Figure 3), the semiconducting properties of TiO_2_ enable photoexcitation under UV or visible light, generating electron–hole pairs that drive the oxidation of glucose, producing a measurable photocurrent proportional to its concentration [51]. Performance enhancements have been achieved through the incorporation of plasmonic metals (e.g., Au, Pt), co-catalysts (e.g., BiOBr, CuO), and heterostructures (e.g., CdS, CdxZn_1−x_S), which extend light absorption into the visible region and improve charge separation. These modifications contribute to improved sensor sensitivity, lower detection thresholds, faster response times, and strong selectivity, even in complex biological environments like blood, sweat, and serum.

Collectively, these sensor modalities highlight the versatility of TiO_2_ nanotubes, offering tailored solutions for selective, stable, and ultrasensitive glucose sensing in diverse environments.

## 4. Application of TiO_2_-Based Sensors in Glucose Biosensing

TiO_2_, particularly in NTA configurations, has emerged as a highly promising platform for glucose biosensing due to its unique physicochemical properties such as high surface area, biocompatibility, photoactivity, and ease of functionalization. This section categorizes and discusses various TiO_2_-based glucose biosensors according to their operational mechanisms—enzymatic, non-enzymatic, PEC, and other novel formats.

### 4.1. TiO_2_-Based Enzymatic Glucose Biosensors

#### 4.1.1. TiO_2_/GOx-Based Biosensors

Recent progress in TiO_2_ NTA-based enzymatic glucose biosensors reflects a growing emphasis on fine-tuning the nanostructure–enzyme interface to enhance sensitivity, selectivity, and operational robustness. Wang et al. [53] addressed the challenge of preserving the intrinsic properties of TiO_2_ NTAs by developing a biosensor in which GOx was immobilized via a carefully optimized cross-linking method that deliberately excluded hybrid material integration. The vertically aligned TiO_2_ NTAs, anodically grown on titanium substrates, ensured strong adhesion, uniform morphology, and high surface area for enzyme loading. The resulting biosensor exhibited a broad dynamic range, ultra-low detection limit, and strong resistance to electrochemical interference from ascorbic acid and sucrose, which are commonly encountered in biological matrices. Importantly, the sensor demonstrated excellent reproducibility and retained functional stability during long-term storage, underscoring the value of structural preservation in enzyme–nanotube coupling.

Building on this, Hu et al. [54] tackled the conductivity bottleneck and enzyme immobilization inefficiencies by introducing a defect-engineering strategy through thermal annealing in an inert argon atmosphere. This process generated oxygen vacancies within the TiO_2_ lattice, enhancing both surface reactivity and charge carrier density, which in turn improved enzyme anchoring and electron transfer kinetics. GOx-functionalized NTAs derived from this process displayed significantly enhanced electrochemical responses, with high sensitivity, a lowered detection threshold, and excellent selectivity toward glucose in the presence of common interferents. The authors emphasized that their method is not only effective but also scalable and economically viable, making it suitable for large-scale biosensor fabrication. Unlike Wang et al.’s [53] structurally conservative approach, Hu et al. [54] introduced deliberate lattice-level modifications to tailor the physicochemical landscape of TiO_2_, reflecting a shift from passive immobilization to active structural optimization.

**Figure 4:**
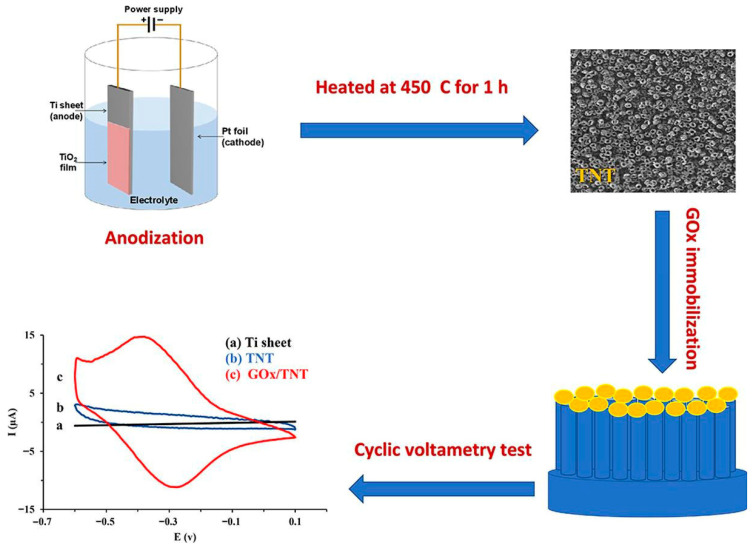
Schematic illustration of the one-step immobilization process of GOx onto TiO_2_ NTAs, enabling the development of a sensitive and efficient glucose biosensor. (Reproduced with permission from [55]).

Akhbari Varkani et al. [55] framed their work within a broader context of integrating redox-active biocatalysts with engineered nanostructures for advanced biosensing applications (Figure 4). They reaffirmed that enzyme immobilization is not only a stability-enhancing step but also a functional determinant of electrocatalytic performance. Employing electrochemically anodized TiO_2_ NTAs, they immobilized GOx to construct a biosensing platform that demonstrated high sensitivity, low detection limits, and an extended linear range for glucose detection. Their results confirmed that TiO_2_ NTAs serve as excellent supports due to their high surface area, biocompatibility, and ability to retain enzyme activity over prolonged use.

Together, these studies highlight the evolving design philosophy in TiO_2_-based biosensor research: from minimalistic structural preservation to defect-driven performance enhancement and finally to system-level integration of nanomaterials with bioactive components.

#### 4.1.2. TiO_2_/Noble Metal/GOx-Based Hybrid Biosensors

Recent developments in TiO_2_ NTA-based enzymatic glucose biosensors reflect a systematic evolution in design strategies that leverage noble metals, redox mediators, and conductive nanomaterials to enhance electrochemical response, enzyme immobilization efficiency, and transduction performance.

Benvenuto et al. [56] developed a high-performance biosensor by sequentially modifying TiO_2_ NTAs with a Au layer via argon plasma treatment, followed by electrodeposition of PB, a known artificial peroxidase. Subsequently, GOx was co-immobilized with chitosan on the PB/Au-modified surface, which provided a redox-active and biocompatible matrix facilitating effective enzyme anchoring and electron mediation. The resulting biosensor exhibited high electrocatalytic activity, a significantly reduced detection limit, and excellent operational stability under repetitive use, with negligible signal drift over extended periods. Notably, the integration of PB facilitated direct electron transfer pathways between GOx and the electrode surface, while the Au layer improved conductivity, collectively enhancing the sensor’s responsiveness and durability under physiological conditions.

Extending the nanocomposite strategy, Feng et al. [57] reported the fabrication of a GOx-based biosensor through the immobilization of GOx onto TiO_2_ NTAs modified with photoreduced Ag nanoparticles (AgNPs). The AgNPs, formed by immersing the TiO_2_ arrays in silver nitrate solution under controlled photoreduction conditions, exhibited uniform distribution and tunable size and density depending on the immersion duration. This metallic decoration significantly enhanced the catalytic surface and electron transfer kinetics, leading to improved amperometric sensitivity and a linear detection range compatible with physiological glucose concentrations. The study demonstrated that precise control over AgNP distribution plays a pivotal role in optimizing sensor performance without compromising the structural integrity of the TiO_2_ substrate.

**Figure 5:**
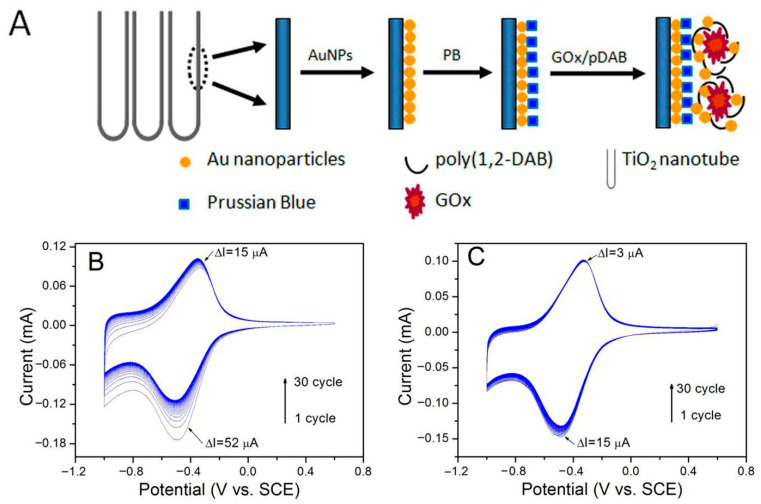
(**A**) Schematic representation of the fabrication procedure for the “bienzyme” electrode based on TiO_2_ NTAs. (**B**) Cyclic voltammograms of the PB/AuNP/TiNT electrode and (**C**) the pDAB-PB/AuNP/TiNT nanocomposite electrode, measured in air-saturated 0.01 M phosphate-buffered saline (PBS, pH 6.0) at a scan rate of 50 mV s^−1^. (Reproduced with permission from [58]).

Building on the role of photoactivity in TiO_2_, Gao et al. [58] employed a photocatalytic deposition technique to embed PB nanocrystals within the inner walls of the TiO_2_ nanotubes, utilizing UV illumination to achieve spatially controlled and stable incorporation (Figure 5). Following this, a polymer-assisted electrodeposition process was used to immobilize GOx in conjunction with AuNPs, creating a nanobiocomposite interface with enhanced electrocatalytic properties. The fabricated bioelectrode demonstrated a rapid and stable amperometric response to glucose, with a broad linear detection range and high reproducibility. The synergy between the peroxidase-mimicking PB, the conductive AuNPs, and the high-surface-area TiO_2_ NTAs enabled efficient electron transfer and preserved enzyme activity, making the system highly suitable for continuous monitoring applications. Furthermore, the spatially resolved functionalization method illustrated a scalable pathway for tailoring the internal architecture of NTAs to host multiple bioactive and redox-active components.

In a distinct approach, Pang et al. [59] engineered a hybrid TiO_2_/CNT electrode by incorporating vapor-grown carbon nanotubes into the internal channels of the TiO_2_ NTAs to address inherent limitations in charge transport and active surface area. This CNT integration dramatically improved electrical conductivity and enzyme loading capacity due to the interconnected nanotubular structure and high aspect ratio of CNTs. Pt NPs were subsequently deposited onto the TiO_2_/CNT composite, forming a uniform catalytic layer optimized for H_2_O_2_ oxidation, a key intermediate in GOx-mediated glucose sensing. GOx immobilization on this multifunctional composite yielded a biosensor characterized by a fast electrochemical response, high sensitivity across physiologically relevant glucose concentrations, and an exceptionally low detection limit. The hybrid configuration demonstrated superior electron mobility and catalytic efficiency compared to conventional TiO_2_-based electrodes, confirming the critical role of CNTs and Pt NPs in advancing sensor performance.

Together, these studies exemplify the progression in biosensor engineering from single-metal nanoparticle decoration to complex multi-component nanocomposite architectures. The integration of noble metals (Ag, Au, Pt), redox mediators (PB), and conductive frameworks (CNTs) into TiO_2_ NTAs has been shown to address the multifaceted challenges of electron transfer, catalytic efficiency, and enzyme stability.

### 4.2. TiO_2_-Based Non-Enzymatic Glucose Biosensors

#### 4.2.1. TiO_2_/Noble Metal-Based Biosensors

Recent developments in non-enzymatic glucose detection have emphasized the integration of noble metals with TiO_2_ nanostructures to enhance electrocatalytic activity, electron transport efficiency, and sensor reusability. TiO_2_-based hybrid platforms modified with noble metals have therefore attracted considerable attention due to their synergistic physicochemical and electrochemical properties.

Song et al. [60] systematically demonstrated the fabrication and electrochemical application of Pt-modified TiO_2_ NTAs as a reusable sensor for glucose detection. In this architecture, Pt nanoparticles functioned dually as catalytic agents for glucose oxidation and as conductive facilitators, enhancing electron mobility along the TiO_2_ framework to support stable amperometric operation. Importantly, the intrinsic photocatalytic functionality of TiO_2_ was preserved, allowing for regeneration of Pt catalytic activity via photodegradation of surface-bound interfering species. This configuration enabled selective, reusable glucose sensing with minimal interference from common electroactive compounds.

In a subsequent study, Wang et al. [61] introduced a bimetallic modification approach by depositing hollow Ag and Pt nanostructures onto annealed TiO_2_ nanotube substrates using a solution-phase reduction and galvanic replacement technique. The resulting Ag&Pt-TiO_2_ composites exhibited well-defined hollow morphologies with uniform nanoparticle distribution, as verified by electron microscopy and elemental mapping. Electrochemical analysis revealed enhanced redox activity and rapid electron transfer kinetics, and demonstrated high sensitivity, wide linear range, and low detection threshold for glucose. This dual-metal TiO_2_-based sensor offered a simplified synthesis route with superior analytical performance.

#### 4.2.2. TiO_2_/Transition Metal-Based Biosensors

##### TiO_2_/Nickel-Based Biosensors

Transition metal-functionalized TiO_2_ nanostructures have garnered increasing interest in non-enzymatic glucose sensing due to their cost-effectiveness, abundance, and catalytic redox activity. Among these, nickel-based modifications of TiO_2_ nanotubes have shown considerable promise.

Yu et al. [62] designed a non-enzymatic glucose sensor using TiO_2_ NTAs decorated with nickel nanoparticles, synthesized via anodization followed by pulsed electrodeposition. Structural characterizations confirmed the uniform incorporation of spherical Ni nanoparticles within the nanotube matrix. Electrochemical assessments demonstrated substantial catalytic activity for glucose oxidation, attributed to the enlarged electroactive surface area and enhanced charge transfer enabled by the nanotubular architecture. The sensor exhibited high sensitivity, a broad linear detection range, and a low detection threshold. Importantly, the straightforward and cost-effective synthesis protocol underscored its feasibility for scalable manufacturing.

**Figure 6:**
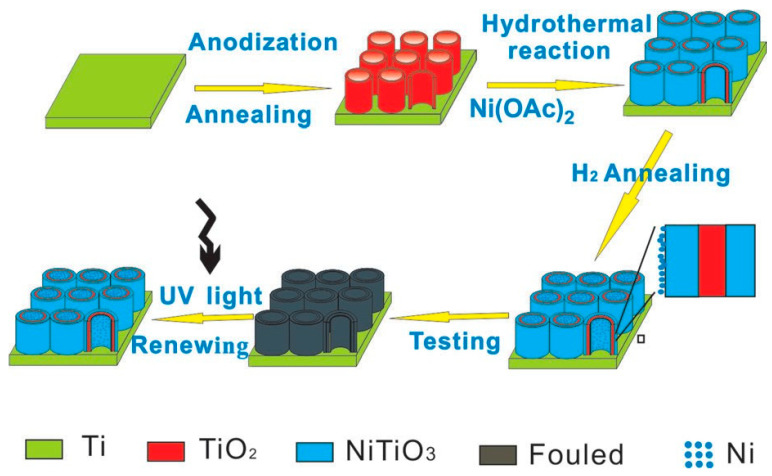
Schematic illustration of the fabrication procedure for the Ni/NiTiO_3_/TiO_2_ electrode and the photoinduced regeneration mechanism of its surface. (Reproduced with permission from [63]).

Expanding upon the catalytic functionality, Huo et al. [63] introduced a light-renewable TiO_2_-based sensor composed of Ni/NiTiO_3_-modified TiO_2_ NTAs fabricated via hydrothermal treatment in nickel acetate, followed by annealing in a reducing atmosphere. This electrode architecture combined the redox-active Ni/NiTiO_3_ phases with the inherent photocatalytic properties of TiO_2_, yielding a sensor capable of self-regeneration under light irradiation (Figure 6). The vertically aligned nanotubes and uniformly distributed active phases facilitated rapid electron transfer and superior electrocatalytic performance at low applied potential. Notably, the photocatalytic restoration of electrode sensitivity after fouling events represented a substantial advancement toward achieving long-term stability in non-enzymatic glucose biosensing.

Kang et al. [64] pursued an alternative strategy by incorporating nickel and diamond-like carbon (DLC) into TiO_2_ NTAs through anodization, electrodeposition, and magnetron sputtering. Electrochemical evaluation revealed markedly improved glucose oxidation performance compared to bare and Ni-coated TiO_2_ electrodes. The enhanced activity was linked to the synergistic interaction between the redox-active Ni species and the conductive DLC layer, which collectively enhanced charge mobility and surface reactivity. The resulting sensor demonstrated high sensitivity and low detection limits across a broad linear range.

##### TiO_2_/Copper-Based Biosensors

The integration of Cu-based nanostructures with TiO_2_ NTAs has been widely explored to enhance the electrochemical performance of non-enzymatic glucose biosensors. Luo et al. [65] developed a CuO-functionalized TiO_2_ nanotube electrode using a two-step process involving electrodeposition of Cu nanoparticles followed by thermal oxidation. The fabricated electrode demonstrated significant electrocatalytic efficiency in alkaline media, exhibiting strong linearity and high sensitivity in glucose oxidation. Their design strategy effectively minimized common interferences and ensured electrode stability over prolonged periods. They concluded that the structural synergy between CuO and TiO_2_ nanotubes was key to the observed selectivity and operational robustness.

In another study, Stanley et al. [66] modified vertically aligned TiO_2_ nanotubes with CuO mesoclusters through electrochemical methods. Their surface morphology analysis revealed well-distributed CuO aggregates that enhanced the catalytic surface area. The electrode facilitated glucose oxidation at a relatively higher potential and achieved remarkable sensitivity over a wide concentration range. The system displayed negligible cross-reactivity with interfering biomolecules and sugars, ensuring reliable performance in complex biological samples. The authors further validated its clinical potential through successful testing with real blood serum, aligning well with commercial glucose sensing tools.

Bhanu et al. [67] introduced a UV-assisted photoreduction route for depositing Cu nanostructures on anodically synthesized TiO_2_ nanotubes. Their approach ensured uniform surface coverage and optimal electronic properties, confirmed by comprehensive structural and optical analyses. The Cu-modified electrodes demonstrated enhanced sensitivity and a broad detection range, with significant improvement in signal selectivity. Moreover, the hybrid structures exhibited superior photocurrent response, indicating dual functionality in both sensing and solar-driven electrochemical applications. They further emphasized that the incorporation of copper not only improved glucose detection but also opened pathways for multifunctional device development.

##### TiO_2_/Transition Metals Hybrid-Based Biosensors

TiO_2_ nanotube-based hybrid structures with transition metals have emerged as effective platforms for non-enzymatic glucose sensing due to their unique electrochemical characteristics and tunable surface functionalities.

Li et al. [68] synthesized Ni-Cu nanoparticles on TiO_2_ NTAs using a potential step method to improve non-enzymatic glucose detection. Surface morphology and composition analysis confirmed uniform alloy distribution with enhanced electrocatalytic properties. Compared to individual nickel or copper modifications, the bimetallic Ni–Cu system showed superior performance in glucose oxidation under alkaline conditions. Interference studies indicated minimal signal distortion from biologically relevant molecules. The sensor also exhibited strong operational stability and repeatability, highlighting its application in complex biological systems.

A sensor was fabricated by Suneesh et al. [69] via electrodepositing Co-Cu alloy nanoparticles onto vertically aligned TiO_2_ NTAs. The electrodeposition parameters were optimized to tailor the alloy composition for maximum glucose oxidation activity. The resulting nanostructured electrode demonstrated dual linear detection ranges with outstanding sensitivity and low detection thresholds. Selectivity tests revealed robust resistance against common electroactive species and sugars. The system was successfully applied for serum glucose estimation, showcasing clinical applicability.

Chen et al. [70] employed polydiallyldimethylammonium chloride (PDDA) to stabilize functionalized TiO_2_ nanotubes, enabling uniform self-assembly of Pd nanoparticles on the electrode surface. The resulting sensor demonstrated enhanced electrocatalytic oxidation of glucose at low potential, maintaining high resistance to interference from common biological species. Surface treatment with acid and base further amplified the glucose oxidation current, attributed to increased surface defects and oxide species. These modifications significantly improved the sensor’s performance within physiologically relevant glucose concentrations.

**Figure 7:**
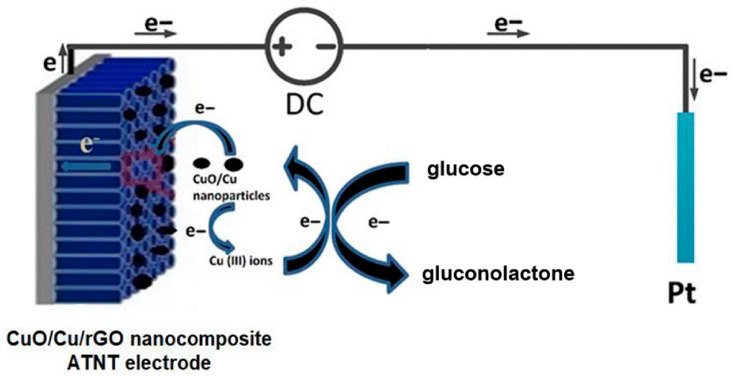
Electrochemical mechanism of glucose sensing by the CuO/Cu/rGO nanocomposite ATNT electrode at an applied potential of 0.6 V. (Reproduced with permission from [71]).

In another study, Chahrour et al. [71] introduced a hybrid nanocomposite comprising CuO/Cu and reduced graphene oxide on anodically formed TiO_2_ NTAs (Figure 7). The stepwise electrodeposition and reduction processes facilitated uniform nanoparticle dispersion and enhanced conductivity through graphene integration. Structural and elemental characterization verified effective composite formation with improved electrochemical surface properties. The biosensor delivered a high electrocatalytic response to glucose with excellent selectivity in neutral media. Its reproducibility and long-term stability emphasize its promise for biomedical diagnostics and pharmaceutical screening.

Kumar & Sinha [72] developed a tungsten oxide-decorated TiO_2_ NTA through electrochemical methods to enhance glucose detection without enzymes. The integration of WO_3_ nanostructures provided a high electroactive surface area and improved stability under electrochemical conditions. Detailed structural analyses confirmed uniform deposition and strong crystalline features of the hybrid material. Electrochemical testing revealed excellent sensitivity and response characteristics, indicating efficient electron transport and charge transfer kinetics. The sensor exhibited consistent behavior in real sample analysis, validating its potential for practical glucose monitoring.

Non-enzymatic glucose biosensors based on TiO_2_ nanostructures have advanced through integration with noble and transition metals to enhance catalytic activity, conductivity, and stability. Noble metal hybrids (Pt, Ag, Au) improved electron transfer and reusability, while transition metal systems (Ni, Cu, Co, Pd, W) offered cost-effective, redox-active alternatives with strong sensitivity and durability. Bimetallic and hybrid designs further boosted selectivity and operational stability, underscoring a clear shift from enzyme-reliant to catalyst-driven TiO_2_ platforms for reliable and scalable glucose detection.

### 4.3. TiO_2_-Based Photoelectrochemical Glucose Biosensors

The development of TiO_2_-based PEC glucose biosensors has attracted increasing attention due to their high sensitivity, stability, and potential for non-invasive diagnostics.

#### 4.3.1. TiO_2_/Noble Metal-Based Biosensors

Noble metal-functionalized TiO_2_-based PEC biosensors have gained significant attention due to their enhanced charge separation, plasmonic light absorption, and catalytic properties. The incorporation of metals such as Au and Pt onto TiO_2_ nanotube architectures has enabled the development of highly sensitive and selective glucose sensing platforms with superior PEC performance.

Liu et al. [73] reported the fabrication of a PEC glucose sensor utilizing TiO_2_ nanotubes functionalized with Au nanoparticles, capitalizing on the superior charge carrier separation efficiency intrinsic to one-dimensional architectures and the pronounced surface plasmon resonance (SPR) properties of Au. The sensor was responsive to visible red-light irradiation, enabling efficient PEC activity. Under optimized operational parameters, the Au/TiO_2_NTs-based sensor demonstrated high sensitivity and an exceptionally low detection threshold for glucose quantification. Furthermore, it maintained robust selectivity, exhibited substantial resistance to interference from coexisting analytes, and preserved functional stability over extended periods. Owing to these advantageous attributes, the system was identified as a promising candidate for integration into non-invasive PEC biosensing platforms.

**Figure 8:**
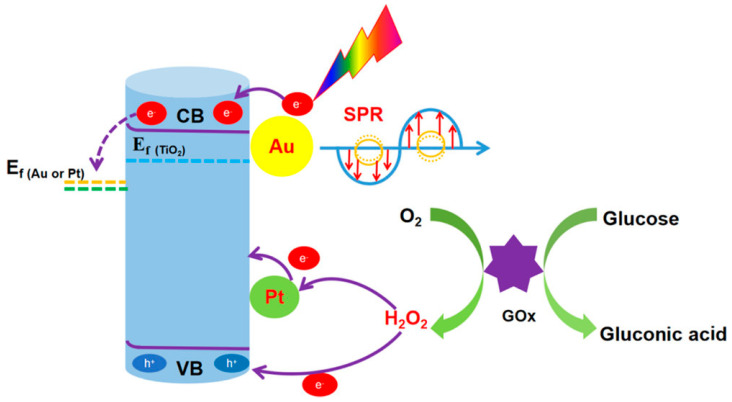
Photoelectrochemical sensing mechanism of the TiO_2_NTs/Au/Pt/GOx biosensor for glucose detection. (Reproduced with permission from [74]).

Yang et al. [74] developed a high-performance PEC glucose biosensor by modifying TiO_2_ NTAs with Au and Pt nanoparticles (TiO_2_NTs/Au/Pt/GOx). The TiO_2_ nanotubes enhanced charge transport by aligning light absorption with carrier diffusion (Figure 8). Au nanoparticles improved light harvesting via plasmonic effects and promoted charge separation through Schottky junctions, while Pt nanoparticles catalyzed hydrogen peroxide reduction from the GOx-mediated glucose reaction, enhancing electron transfer. This dual modification significantly outperformed single-metal systems, demonstrating that Pt plays a critical role in boosting sensitivity and detection efficiency in enzymatic PEC glucose sensing.

#### 4.3.2. TiO_2_/Metal Oxide-Based Biosensors

The integration of TiO_2_ with metal oxides has emerged as a strategic approach to enhance PEC glucose sensing by leveraging synergistic heterojunction effects and catalytic functionalities. These composite systems enable improved charge separation, broadened light absorption, and enzyme-free or self-powered detection capabilities, broadening the scope of non-invasive biosensing applications.

**Figure 9:**
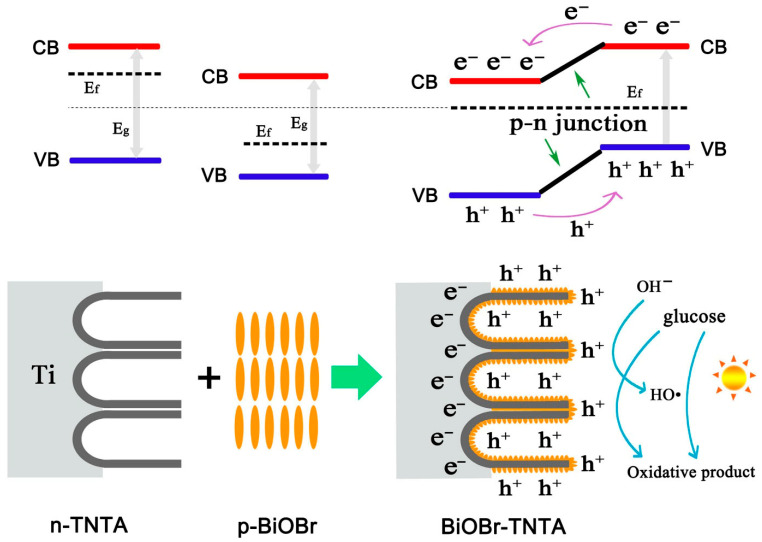
Illustrative representation of the photoelectrochemical glucose oxidation mechanism at the BiOBr-TNTA electrode. (Reproduced with permission from [75]).

Wu et al. [75] engineered a p-n heterojunction PEC electrode by depositing BiOBr nanostructures onto TiO_2_ NTAs (Figure 9). The heterojunction structure facilitated effective photogenerated charge separation due to favorable energy band alignment between BiOBr and TiO_2_. The electrode preparation parameters, including the Bi precursor concentration and nanotube morphology, critically influenced the sensor’s electrochemical properties. Under optimized conditions, the device displayed enhanced response and reproducibility for glucose detection in alkaline media. This study expanded the material design strategy for PEC biosensors beyond traditional semiconductors.

Ke et al. [76] critically addressed the limitations posed by conventional invasive glucose sensors, which often resulted in adverse physical and psychological effects during fingertip blood sampling, constrained applicability of enzyme-dependent electrodes, and operational complexities associated with externally powered integrated systems. In response, they proposed a PEC platform capable of enzyme-free, self-powered glucose detection via sweat, utilizing a CuO nanoparticle-modified TiO_2_ hierarchical nanotube (CuO\@TiO_2_ HNT) structure. The CuO\@TiO_2_ HNT-based PEC sensor demonstrated reliable glucose detection under bias-free conditions and maintained functional integrity across a wide thermal operating range. Analytical performance for sweat glucose quantification before and after food intake showed strong agreement with results obtained from standard commercial glucometers. The superior sensing capability was primarily attributed to the enhanced photon absorption and high surface area provided by the hierarchical TiO_2_ nanotube framework, in conjunction with the CuO nanoparticles’ ability to accelerate interfacial charge transfer and promote glucose oxidation kinetics. This study introduced a promising self-sustained, non-invasive strategy for sweat-based glucose monitoring, offering a viable route toward the development of portable and enzyme-independent biosensing technologies.

Yang et al. [77] emphasized that achieving elevated sensitivity and minimal detection thresholds represented essential benchmarks in the advancement of biosensing technologies. They synthesized a PEC glucose sensor by employing three-dimensional hydrogen titanate nanotubes grown on titanium substrates as precursors, subsequently forming a CuO–TiO_2_ heterojunction nanotube array (CuO–TiO_2_NTs/Ti) photoelectrode. The integrated architecture, comprising a conductive titanium foil, a mesoporous nanotubular morphology, and a p-n CuO–TiO_2_ interface, markedly enhanced charge carrier dissociation efficiency and promoted rapid interfacial electron transport, resulting in amplified photoelectronic responses under visible light exposure. The sensor exhibited high analytical sensitivity, low detection thresholds, and notable selectivity in glucose recognition. Density functional theory calculations confirmed the role of the heterojunction in improving photoinduced electron–hole separation and overall PEC performance. Furthermore, the incorporation of chemometric methodologies significantly optimized signal interpretation, thereby advancing the precision of sensitivity measurements and reinforcing the practical viability of the PEC sensor in bioanalytical applications.

#### 4.3.3. TiO_2_/Semiconductor-Based Biosensors

The formation of heterojunctions between TiO_2_ and narrow band gap semiconductors has emerged as an effective approach to enhance PEC glucose sensing by promoting light absorption and charge carrier separation. Such TiO_2_-based semiconductor composites offer enzyme-free detection capabilities with improved sensitivity, selectivity, and practical applicability in clinical diagnostics.

Liu et al. [78] reported that a composite structure integrating g-C_3_N_4_ and TiO_2_ nanosheets was strategically engineered to construct a photoelectrochemical enzyme biosensor platform. The coupling of these semiconductors was designed to overcome the limited visible-light activity of TiO_2_ and to suppress the recombination of photogenerated charge carriers within g-C_3_N_4_, thereby improving overall photoelectrochemical efficiency. Employing glucose oxidase as the biorecognition element, the hybrid system exhibited pronounced visible-light-driven catalytic behavior toward enzymatic glucose oxidation, surpassing the performance of biosensors fabricated from the individual constituents. The enhanced activity was attributed to the synergistic heterojunction effect that facilitated efficient charge transfer, as well as to the high surface area and biocompatible characteristics of the TiO_2_ nanosheets, which collectively promoted superior enzyme immobilization and signal transduction within the photoelectrochemical sensing interface.

Esmaeili et al. [79] designed an enzyme-free PEC glucose sensor by decorating TiO_2_ NTAs with Cd_x_Zn_1−n_S nanofilms. This heterojunction composite structure significantly improved light absorption and electron transport, resulting in amplified photocurrent signals. The modified electrode showed excellent analytical performance with respect to detection limit and sensitivity, attributed to the discrete morphology of the sulfide films. Characterization confirmed the successful integration of the composite and its structural stability. The sensor further demonstrated robust application potential in detecting glucose in real plasma samples, underscoring its clinical relevance and economic viability.

Li et al. [80] demonstrated that heterojunctions composed of dissimilar semiconducting materials exhibited superior photon-harvesting capabilities and enhanced photoelectrical responses relative to single-component systems. They engineered CdSe/TiO_2_ nanotube (CdSe/TiO_2_NT) heterojunctions via a hydrothermal synthesis route, utilizing structurally stable TiO_2_ nanotubes as the foundational scaffold. The resultant nanostructures were extensively characterized through X-ray diffraction, transmission and scanning electron microscopy, photoluminescence spectroscopy, UV–visible absorption spectroscopy, and electrochemical impedance spectroscopy. Under visible light excitation, the CdSe/TiO_2_NT heterojunctions displayed enhanced optical absorption and a significant reduction in charge carrier recombination compared to pristine TiO_2_. Theoretical simulations substantiated the effective spatial separation of photogenerated electrons and holes within the heterostructure. When optimized, the PEC system demonstrated a linear photocurrent response across a defined glucose concentration range and maintained a low detection threshold. Additionally, the sensor showed high selectivity and operational stability. The underlying PEC sensing mechanism was elucidated by integrating empirical observations with theoretical analyses, reinforcing the potential of CdSe/TiO_2_-based heterostructures in advanced biosensing platforms.

#### 4.3.4. TiO_2_/Graphene-Based Biosensors

**Figure 10:**
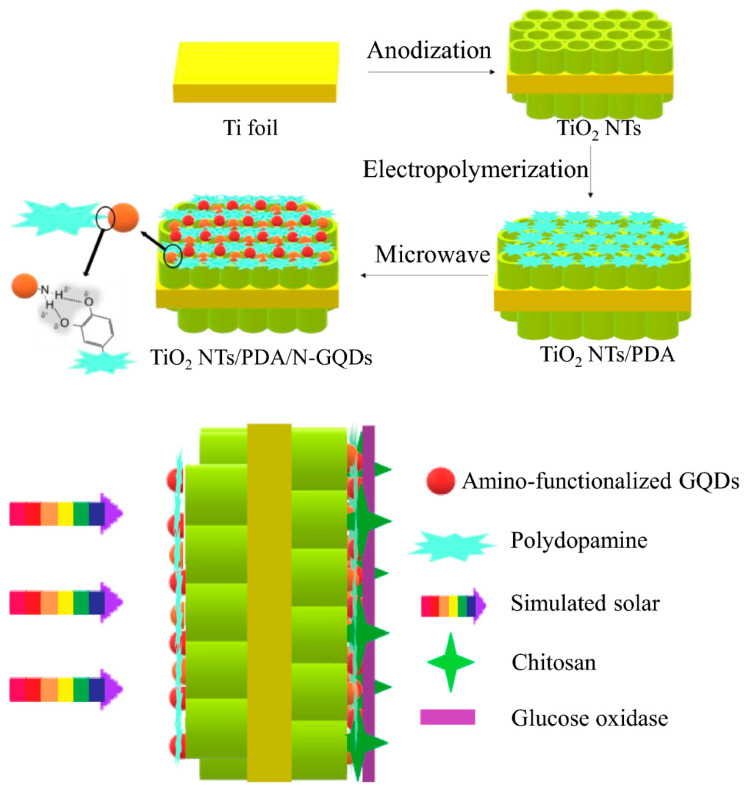
The fabrication process of the TiO_2_ NTs/PDA/N-GQD dual-electron-acceptor biosensing platform. (Reproduced with permission from [81]).

The integration of graphene-based nanomaterials with TiO_2_ has shown great promise in enhancing PEC biosensing performance through improved electron transfer and charge separation. Such hybrid architectures enable high photocurrent responses and reliable glucose detection, highlighting their potential for advanced biosensor development. Yang et al. [81] fabricated a PEC biosensor by functionalizing TiO_2_ nanotubes with polydopamine and amino-functionalized graphene quantum dots, integrated with GOx (Figure 10). The electropolymerized polydopamine provided an efficient electron-transfer interface, while the microwave-assisted incorporation of quantum dots improved charge carrier separation. The system demonstrated notable enhancement in photocurrent generation and exhibited superior analytical performance in terms of selectivity and operational stability. This dual-electron-acceptor design indicated the potential of synergistic material integration in advancing PEC biosensor platforms.

TiO_2_-based PEC glucose biosensors have progressed through integration with noble metals, metal oxides, semiconductors, and graphene to improve charge separation, light absorption, and catalytic efficiency. Noble metals enhanced plasmonic response and electron transfer, while metal oxides and semiconductors enabled enzyme-free, self-powered detection with high sensitivity and stability. Graphene hybrids further boosted conductivity and photocurrent. Overall, these developments reflect a move toward light-driven, wearable PEC sensors for practical glucose monitoring.

### 4.4. TiO_2_-Based Other Novel Glucose Biosensors

Beyond traditional electrochemical and PEC designs, novel TiO_2_-based biosensors have employed organic electrochemical transistors (OECTs) and optical detection strategies for glucose monitoring.

Liao et al. [82] developed an organic electrochemical transistor (OECT) sensor incorporating TiO_2_ NTAs as the gate electrode, replacing traditionally used Pt-based materials. The TiO_2_ NTAs offered comparable electrocatalytic performance while being cost-effective and biocompatible. When modified with Pt nanoparticles and GOx, the device demonstrated a strong linear correlation between glucose concentration and output signal, along with enhanced selectivity against common interferents through Nafion functionalization. Furthermore, the sensor maintained stable signal reproducibility, indicating the robustness of TiO_2_ NTAs as an alternative gate material in OECT configurations. This approach suggested significant potential for expanding OECT-based biosensing technologies using TiO_2_ nanostructures.

**Figure 11:**
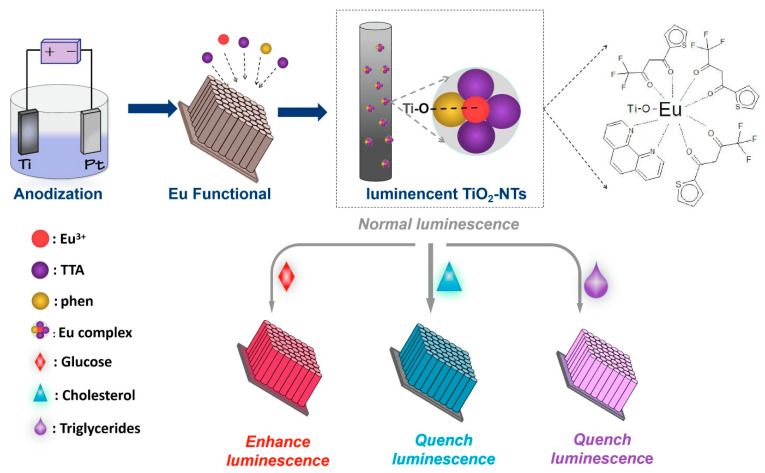
Schematic representation depicting the fabrication of Eu(III) complex-functionalized TiO_2_ NTAs and their fluorescence-based sensing mechanism for glucose, cholesterol, and triglycerides. (Reproduced with permission from [83]).

Building upon the multifunctionality of TiO_2_ NTAs, Su et al. [83] introduced a luminescent sensor platform by integrating europium complexes onto TiO_2_-nanotubes through a one-step anodization and in-situ modification technique (Figure 11). The europium-functionalized nanotubes exhibited distinct fluorescence behavior upon interaction with glucose, cholesterol, and triglycerides, which was attributed to an energy transfer mechanism. A linear response in fluorescence intensity relative to the analyte concentration allowed quantitative detection with high sensitivity. This photoluminescent sensor was further validated through practical applications in food matrices, exhibiting strong agreement with certified analytical methods. The study highlighted the potential of rare-earth-doped TiO_2_ nanostructures for constructing simple, selective, and multifunctional biosensing platforms, expanding the utility of TiO_2_-based systems in real-world scenarios.

The prior discussion of TiO_2_ NT-based biosensing platforms for glucose detection is summarized in Table 1.

## 5. Challenges and Future Perspectives

Despite significant advancements in TiO_2_-based glucose sensor technologies, several critical challenges hinder their broader clinical translation, long-term operation, and scalability. Enzymatic sensors, while achieving prolonged stability and fast response times, face limitations due to the inherent instability of GOx, which is prone to denaturation under varying pH, temperature, and storage conditions. Although studies have demonstrated operational stability exceeding 30 days and response times under one second, issues such as enzyme leaching and biofouling in complex biofluids like serum persist, restricting their use in continuous monitoring applications. Non-enzymatic sensors, particularly those incorporating transition metals or bimetallic nanostructures, offer superior electrocatalytic activity, wide linear ranges, and ultra-low detection limits. However, their reliance on strongly alkaline media raises compatibility concerns with physiological fluids and wearable applications. Additionally, despite improved selectivity through nanostructuring, interference from electroactive species in high-protein environments remains problematic. Challenges such as nanoparticle agglomeration, structural instability, and limited shelf life further impede their commercial viability. PEC sensors, exploiting TiO_2_’s semiconductor properties under light illumination, present a promising alternative with advantages like low-power operation, high sensitivity, and background current suppression. Systems incorporating materials such as CdxZn_1−x_S and BiOBr have achieved remarkably low detection limits, yet challenges persist in optimizing light-harvesting efficiency, particularly for visible-light-driven PEC sensors. Ensuring stable performance under fluctuating ambient lighting and standardizing optical setups remain unresolved issues, complicating their integration into compact, real-world devices. Another critical gap lies in real-world validation, as many studies focus on performance in controlled buffer solutions rather than complex biological matrices like serum or plasma. The lack of extensive clinical trials, long-term monitoring studies, and standardized fabrication methods further delays their transition from lab-scale prototypes to commercial products. Table 2 highlights the features, advantages, and limitations of enzymatic, non-enzymatic, and PEC TiO_2_-based glucose sensors.

Future research must prioritize hybrid sensor architectures that combine the selectivity of enzymatic recognition with the robustness of non-enzymatic and PEC systems, potentially enabling dual-signal amplification for enhanced reliability. The development of flexible, wearable TiO_2_-based sensors is essential for continuous glucose monitoring, requiring innovations in biocompatible materials and stretchable substrates to improve user comfort and signal stability. Integrating advanced data processing tools such as machine learning [84] and artificial intelligence [85] could further refine dynamic calibration, drift correction, and predictive analytics, enabling personalized glucose monitoring tailored to individual metabolic patterns. Scalable and cost-effective fabrication techniques, including roll-to-roll printing [86] and 3D nanostructuring [87], are needed to replace lab-intensive synthesis methods and facilitate mass production. Additionally, sustainability considerations, such as eco-friendly synthesis routes, reduced use of rare metals, and recyclable materials, must be incorporated to align with green chemistry principles. Finally, rigorous clinical validation, long-term in vivo studies, and adherence to regulatory standards are imperative to bridge the gap between research and practical implementation, ensuring these sensors meet the demands of point-of-care diagnostics and global healthcare markets.

## 6. Conclusions

In conclusion, TiO_2_-based glucose sensors have demonstrated remarkable potential across enzymatic, non-enzymatic, PEC, and emerging hybrid platforms. Their tunable surface chemistry, excellent biocompatibility, and compatibility with a wide range of nanostructures enable sensitive, selective, and stable glucose detection in both physiological and complex real-world matrices. The reviewed literature highlights significant advancements in detection range, response time, and anti-interference capabilities, achieved through strategic material modifications such as metal nanoparticle doping, heterojunction formation, and surface functionalization. Despite these achievements, key challenges remain in long-term stability, reproducibility, and scalability. Recent developments in wearable biosensors based on TiO_2_ nanostructures have further expanded their applicability toward continuous, non-invasive glucose monitoring. Such devices offer distinct advantages, including real-time data acquisition, user convenience, and integration with wireless or smartphone-based platforms for personalized healthcare. Future progress will hinge on the integration of smart data analytics, sustainable material strategies, and wearable device architectures. In this context, designing flexible, skin-compatible, and energy-efficient TiO_2_-based wearable systems represents a promising direction for next-generation biosensing technologies. Collectively, these innovations hold the promise of transforming TiO_2_-based glucose sensors from laboratory prototypes into clinically relevant, real-time monitoring tools capable of addressing the growing demands of personalized and point-of-care diagnostics.

## Figures and Tables

**Table 1:** Key parameters of TiO_2_ NT-based biosensing platforms for glucose detection.

Biosensor Configuration	Biosensor Type	Target	Medium Used	Primary Detection Method	Linear Range	Sensitivity	Detection Limit	Response Time	Stability	Anti-Interference Performance	Reference
TiO_2_/GOx	Electrochemical (Enzymatic)	Glucose	PBS	Amperometry	0.05 to 0.65 mM	199.6 μA mM^−1^cm^−2^	3.8 μM	NA	86% (after 6 days)	Minimal response to ascorbic acid, sucrose, L-cysteine, L-histidine, and L-glycine	Wang et al. [53]
TiO_2_/GOx	Electrochemical (Enzymatic)	Glucose	PBS	Amperometry	0.05 to 3.2 mM	8.5 μAmM^−1^cm^−2^	3.2 μM	<10 s	93.5% (after 40 days)	Minimal response to ascorbic acid, uric acid, and dopamine, KCL	Hu et al. [54]
TiO_2_/GOx	Electrochemical (Enzymatic)	Glucose	PBS	Voltammetry	0.03 to 1.0 mM	56.60 µA mM^−1^ cm^−2^	8.5 µM	10 s	90% (after 20 days)	Minimal response to ascorbic acid, acetaminophen, and uric acid	Akhbari Varkani et al. [55]
Ti/TiO_2_/Au/PB/GOx	Electrochemical (Enzymatic)	Glucose	PBS	Amperometry	0.015 to 4.00 mM	36 μA mM^−1^	5 μM	<10 s	90% (after 21 days)	Minimal response to ascorbic acid, uric acid, acetaminophen	Benvenuto et al. [56]
GOx/Ag/TiO_2_	Electrochemical (Enzymatic)	Glucose	PBS	Amperometry	0.1 to 4 mM	0.39 μA mM^−1^ cm^−2^	0.1 mM	40 s	NA	Minimal response to H_2_O_2_	Feng et al. [57]
GOx/Au/pDAB)-PB/AuNP/TiO_2_	Electrochemical (Enzymatic)	Glucose	PBS, Serum	Amperometry	0.01 to 0.70 mM	248 mA M^−1^ cm^−2^	3.2 μM	<1 s	>90% (after 30 days)	Minimal response to ascorbic acid, uric acid, p-acetamidophenol	Gao et al. [58]
TiO_2_/CNT/Pt/GOx	Electrochemical (Enzymatic)	Glucose	PBS	Amperometry	0.006 to 1.5 mM	0.24 μA mM^−1^ cm^−2^	5.7 μM	<3 s	82% (after 30 days)	Not specified	Pang et al. [59]
Pt/TiO_2_	Electrochemical (Non-enzymatic)	Glucose	H_2_SO_4_	Amperometry	NA	NA	NA	NA	Self-cleaning via UV exposure	Minimal response to ascorbic acid, uric acid, and p-acetamidophenol	Song et al. [60]
Ag/Pt-TiO_2_	Electrochemical (Non-enzymatic)	Glucose	PBS	Voltammetry	30 to 180 mM	3.99 μA·cm^−2^·mM^−1^	22.6 μM	NA	NA	Minimal response to Cl ion	Wang et al. [61]
Ni-NPs/TiO_2_	Electrochemical (Non-enzymatic)	Glucose	NaOH solution	Amperometry	0.004 to 4.8 mM	700.2 μA mM^−1^ cm^−2^	2 μM	<5 s	80.3% (after 20 days)	Minimal response to ascorbic acid, uric acid	Yu et al. [62]
Ni/NiTiO_3_/TiO_2_	Electrochemical (Non-enzymatic)	Glucose	Serum	Amperometry	0.005 to 0.5 mM	456.4 μA mM^−1^ cm^−2^	0.7 μM	<5 s	NA	Minimal response to ascorbic acid, uric acid	Huo et al. [63]
Ni-DLC/TiO_2_	Electrochemical (Non-enzymatic)	Glucose	NaOH	Amperometry	0.99 to 22.97 mM	1063.78 μA·mM^−1^·cm^−2^	0.53 μM	<5 s	82.6% (after 30 days)	Minimal response to dopamine, ascorbic acid, uric acid, and galactose	Kang et al. [64]
CuO/TiO_2_	Electrochemical (Non-enzymatic)	Glucose	Serum	Amperometry	Up to 2.0 mM	79.79 μA·mM^−1^·cm^−2^	1 μM	<4 s	>90% (after 30 days)	Minimal response to Cl ion, ascorbic acid, uric acid, lactose, sucrose, fructose, dopamine	Luo et al. [65]
CuO/TiO_2_	Electrochemical (Non-enzymatic)	Glucose	Serum	Amperometry	0.625 to 6.25 m mol L^−1^; 6.87 to 12.5 m mol L^−1^	1836 μA mmol^−1^ L cm^−2^ (low range); 1416 μA mmol^−1^ L cm^−2^ (high range)	3.4 μ mol L^−1^	≤2 s	>96% (after 30 days)	Minimal response to ascorbic acid, dopamine, galactose, uric acid, lactose	Stanley et al. [66]
Cu/TiO_2_	Electrochemical (Non-enzymatic)	Glucose	NaOH	Amperometry	0.5 to 7 mM	522 μA mM^−1^ cm^−2^	NA	0.1 s	NA	Minimal response to ascorbic acid, NaCl, lactose, sucrose, D-fructose	Bhanu et al. [67]
Ni-Cu/TiO_2_	Electrochemical (Non-enzymatic)	Glucose	NaOH	Amperometry	10 μM to 3.2 mM	1590.9 μA mM^−1^ cm^−2^	5 μM	<5 s	98% (after 49 days)	Minimal response to uric acid, ascorbic acid	Li et al. [68]
Co/Cu/TiO_2_	Electrochemical (Non-enzymatic)	Glucose	Serum, NaOH	Amperometry	Up to 12 mM	4651.0 μA mM^−1^ cm^−2^ up to 5 mM and 2581.70 μA mM^−1^ cm^−2^ from 5 mM to 12 mM.	0.6 μM	NA	92% (after 90 days)	Minimal response to fructose, maltose, galactose, lactose, ascorbic acid, uric acid, acetamidophenol, creatinine, urea, chloride	Suneesh et al. [69]
Pd NPs/PDDA/TiO_2_	Electrochemical (Non-enzymatic)	Glucose	Serum	Amperometry	4 × 10^−7^ to 8 × 10^−4^ M	NA	8 × 10^−8^ M	NA	Stable (after 14 days)	Minimal response to chloride ions, ascorbic acid, uric acid, urea	Chen et al. [70]
CuO/Cu/rGO/TiO_2_	Electrochemical (Non-enzymatic)	Glucose	PBS	Amperometry	0.5 to 16 mM	371.6 μA mM^−1^ cm^−2^	22.8 μM	~5 s	92% (after 5 days)	Minimal response to uric acid, ascorbic acid, lactose, sucrose, fructose	Chahrour et al. [71]
WO_3_/TiO_2_	Electrochemical (Non-enzymatic)	Glucose	Orange juice	Amperometry	1.0 to 6.5 mM	1228.12 μA mM^−1^ cm^−2^	0.19 mM	2 s	97.1% (after 25 days)	Minimal response to uric acid, ascorbic acid, NaCl	Kumar & Sinha [72]
Au/TiO_2_	Photoelectrochemical	Glucose	NaOH	Visible red light	1 to 90 μM	170.37 μA·mM^−1^·cm^−2^	1.3 μM	NA	96% after 25 days	Minimal response to sucrose, lactose, ascorbic acid, saccharose, fructose	Liu et al. [73]
TiO_2_/Au/Pt/GOx	Photoelectrochemical	Glucose	PBS	Amperometry (visible light)	0 to 4 mM	81.93 μA mM^–1^ cm^–2^	1.39 μM	NA	NA	Minimal response to NaCl, sucrose, ascorbic acid, uric acid, galactose, fructose	Yang et al. [74]
BiOBr/TiO_2_	Photoelectrochemical	Glucose	Serum, NaOH)	Amperometry (visible light)	5 × 10^2^ to 3 × 10^7^ nM	NA	10 nM	NA	>95% (after 28 days)	Minimal response to ascorbic acid, uric acid, urea, dopamine	Wu et al. [75]
CuO/TiO_2_	Photoelectrochemical	Glucose	Human sweat	Visible light	1 to 200 μM (sweat)/0.5 to 10 mM (blood)	138.5 μA·mM^−1^·cm^−2^	0.7 μM	<1 s	NA	Minimal response to NaCl, KCl, dopamine, uric acid, lactic acid, ascorbic acid	Ke et al. [76]
3D CuO/TiO_2_/Ti	Photoelectrochemical	Glucose	Serum	Visible light	70 to 900 μM	155 μA·mM^−1^·cm^−2^	20 μM	NA	NA	Minimal response to fructose, lactose, sucrose, dopamine, ascorbic acid, uric acid, and L-cysteine	Yang et al. [77]
GOx/g-C3N4-TiO_2_/ITO	Photoelectrochemical	Glucose	Serum	Visible light	0.05 to 16 mM	16.7 µA mM^−1^ cm^−2^	0.01 mM	NA	90.5% (after 14 days)	Minimal response to ascorbic acid, uric acid, dopamine, fructose, lactose, and sucrose.	Liu et al. [78]
CdxZn1-xS/TiO_2_	Photoelectrochemical	Glucose	Plasma, NaNO_3_	Amperometry (UV light)	0.014 to 214 mM	1331.7 μA mM^−1^ cm^−2^	0.225 μM	NA	82% (after 35 days)	Minimal response to ascorbic acid, uric acid, dopamine, urea, lysine, tyrosine, histidine	Esmaeili et al. [79]
CdSe/TiO_2_	Photoelectrochemical	Glucose	Serum	Visible light	10 to 90 μM	NA	3.1 μM	NA	NA	Minimal response to ascorbic acid, uric acid, urea, fructose, xylose	Li et al. [80]
TiO_2_/PDA/N-GQDs/GOx	Photoelectrochemical	Glucose	PBS	Amperometry (visible light)	Up to 11 mM	13.6 μA mM^−1^ cm^−2^	0.015 mM	<1 s	86.95% (after 30 days)	Minimal response to NaCl, sucrose, ascorbic acid, uric acid, dopamine	Yang et al. [81]
Nafion/GOx/Pt-NPs/TiO_2_	Organic Electrochemical Transistor	Glucose	PBS, Serum	Amperometry	100 nM to 5 mM	0.09 *NCR*/1μM	100 nM	NA	90% (after 10 days)	Minimal response to ascorbic acid, uric acid	Liao et al. [82]
Eu(III) complex/TiO_2_	Fluorescence (Optical)	Glucose	Orange juice	Fluorescence intensity change	0 to 15 mmol/L	NA	1.02 mmol/L	NA	NA	Minimal response to urea, fructose, sucrose, galactose	Su et al. [83]

**Table 2:** Comparison of TiO_2_-based glucose sensor types.

Feature	Enzymatic Sensors	Non-Enzymatic Sensors	Photoelectrochemical (PEC) Sensors
Sensing Principle	Uses an enzyme, such as glucose oxidase (GOx), for specific biorecognition of the target analyte. The biological event is then converted into a measurable signal by a transducer, often electrochemical.	Uses an electrode material (e.g., noble metal, metal oxide, or carbon nanomaterial) to directly catalyze the oxidation or reduction in the target analyte.	Combines light excitation and an electrical signal readout. A photoactive material converts an optical signal into a measurable electrical current or voltage.
Strengths	High selectivity and sensitivity: Enzymes provide excellent specificity for their target molecules, resulting in high accuracy. Rapid response: Enzymatic reactions can provide a fast response time.	High stability and long-term lifespan: Not dependent on biological components, so they are more robust against environmental factors like temperature and pH. Lower cost: Enzymes are often expensive to produce and immobilize, so non-enzymatic sensors have lower manufacturing costs. Simpler fabrication: Avoids the complex and delicate process of immobilizing enzymes.	High sensitivity: Separating the optical excitation and electrical detection minimizes background noise, leading to very high sensitivity and low detection limits. High signal-to-noise ratio: The separation of the input light signal and output electrical signal allows for reduced noise and drift. Low background signal: Inherently low background current enables sensitive detection. Miniaturization: Relies on simple light sources and electrodes, allowing for smaller, more portable devices.
Weaknesses	Low stability: Enzymes can denature due to changes in temperature, pH, or exposure to organic solvents, which reduces sensor lifespan. High cost: Enzymes are costly to source, purify, and immobilize. Complex immobilization: Ensuring the enzyme remains active and securely attached to the electrode is a complicated process. Oxygen dependence: Early generations of sensors were limited by the availability of oxygen, though this has been addressed in later generations.	Lower selectivity: Catalytic materials are not as specific as enzymes, leading to potential interference from other electroactive species in the sample. High working potential: Some non-enzymatic sensors require a high potential, which can increase interference. Surface fouling: The electrode surface can become blocked or “poisoned” by intermediate oxidation products, which degrades performance over time. Sensitivity can be lower: It remains a challenge to achieve sensitivity levels comparable to enzymatic sensors.	Limited material choice: Performance is highly dependent on the photoactive material used, which can have intrinsic limitations like a narrow light absorption range. Complex systems: The use of nanomaterials and heterojunctions can increase complexity, which may affect scalability. Potential for toxicity: Some photoactive materials, like early quantum dots, have toxicity concerns, though research is shifting to more biocompatible options. Developmental stage: PEC technology is still an emerging field, and widespread commercialization is not yet realized for many applications.

## Data Availability

No new data were created or analyzed in this study.
